# Trends in Adverse Childhood Experiences (ACEs) Among Clients of The Exchange Center for Child Abuse Prevention in Alabama's Wiregrass Region

**DOI:** 10.7759/cureus.79553

**Published:** 2025-02-24

**Authors:** Mohkam Singh, Sarah Adkins, Angela Rubino, Aaron Dramann, Mindy Higley, Pamela Miles, Lisa Ennis, Lee Scott

**Affiliations:** 1 Osteopathic Medicine, Alabama College of Osteopathic Medicine, Dothan, USA; 2 Psychology, The Exchange Center for Child Abuse Prevention, Dothan, USA; 3 Library Sciences, Alabama College of Osteopathic Medicine, Dothan, USA; 4 Pediatrics, Alabama College of Osteopathic Medicine, Dothan, USA

**Keywords:** adverse childhood experience, childhood trauma, preventive medicine, rural, trauma-informed care

## Abstract

Adverse childhood experiences (ACEs) are stressful or traumatic events like physical or sexual abuse experienced before the age of 18. ACEs are a national health crisis, with many children in the United States (US) experiencing at least one adverse childhood event. High ACE scores (3+ of 11 categories) are correlated with poor preventable physical and mental health outcomes. Given the unique challenges faced by rural communities, understanding how ACEs uniquely impact rural populations and their health outcomes is imperative to population-specific interventions. The Exchange Center for Child Abuse Prevention (ECCAP), a free non-profit center serving both urban counties (Coffee, Dale, Henry, Covington, Geneva, and Pike) and rural counties (Houston) in Alabama's Wiregrass region, is uniquely poised to serve clients with a history of ACEs. It was hypothesized that ACE scores from ECCAP clients living in rural Wiregrass counties would be higher than those from urban Wiregrass counties. Client data were obtained as a retrospective study using the 10-item ACE survey administered at ECCAP. Participants received trauma-informed therapeutic services through ECCAP from 2019 to 2021 (N=1643). Data collection was done independently by the ECCAP prior to the formation of the hypothesis, which was formed after data collection. Outcome measures were presenting victimization code and ACE scores correlated with the client's county of origin. An overall mean ACE score of 5.897 across seven Wiregrass counties was found, with no significant differences between ACE scores between counties (p>0.05). Exploratory findings indicated that clients who came to the clinic with the in-patient code Adult Sexually Abused as Children (ASAC) had the highest ACE scores of all clients (6.74). This is the first known research to demonstrate high ACE scores across Alabama's Wiregrass counties, where no significant difference was found between scores of clients from urban versus rural counties. This work can benefit the Wiregrass region of Alabama by quantifying the community’s needs, thus demonstrating the importance of trauma-informed centers like ECCAP. Since the population served by the ECCAP is not representative of the population at large, future surveys should sample a broader patient population and delineate urban versus rural trends using zip code rather than county of origin. Future clinical work could integrate ACE surveys into in-patient questionnaire data so clients with high ACE scores can be referred in real-time to evidence-based, trauma-informed services like those provided by ECCAP.

## Introduction

Adverse childhood experiences (ACEs) are stressful or traumatic events experienced before age 18, like physical or sexual abuse. About 45% of children in the United States (US) have experienced at least one ACE [1]. A child’s brain has a high level of plasticity, which allows them to learn quickly and reach developmental milestones, but it can also be affected by traumatic events and stressors. ACEs are commonly associated with the concept of toxic stress.

Toxic stress is the excessive activation of the stress response system in the brain without the ability to escape from the physiological response [2]. Exposure to toxic stress in children and adolescents has been shown to negatively impact their development, both physiologically and behaviorally [3]. Repeated and prolonged exposure to toxic stress dysregulates how the brain would typically respond to the stimuli causing heightened stress hormones and inflammation, resulting in negative health outcomes over time. Socially, ACEs have also been shown to cause a higher likelihood of risk-taking behavior, which may manifest as poor coping mechanisms, a higher potential to fail out of school, and a higher potential to face socio-economic hardships. Higher ACE exposures are specifically linked to smoking, gambling, and alcohol use [3].

ACEs, as toxic stressors, are thought to chronically activate and, therefore, dysregulate the hypothalamic-pituitary-adrenal (HPA) axis, leading to the sustained release of stress-related hormones like cortisol and aldosterone. Concurrently, the sympathetic adrenal medullary (SAM) axis is activated, leading to the release of epinephrine and norepinephrine [4]. The prolonged and excessive activation of the HPA and SAM axes can lead to pathological changes and epigenetic modifications of specific genes [5]. These modifications are often found in "hotspot" genes, altering their expression and function and contributing to long-term health effects [6]. Thus, there exists a strong dose-response association with negative health outcomes as these individuals age; high ACE scores (3+ of 11 categories) are correlated with poor behavioral disorders, diabetes, cardiovascular disease, and other preventable diseases [7-11]. Considering roughly 60% of adults in the US experience at least one ACE in their lifetime, understanding the role of early childhood adversity in the pathogenesis of chronic diseases is critical in the preventive setting [5]. ACE exposure without proper intervention, compounded with additional stressors, can lead to poor social protection, resulting in poor health outcomes for patients [3]. As such, ACE exposures represent a national public health crisis [12]. By identifying exposure to ACEs and the associated toxic stress, patients and clinicians are provided an opportunity for intervention that can drastically improve outcomes.

Given that 14% of the US population resides in rural areas [13], it is crucial to understand how ACEs affect the health outcomes of these communities and tailor interventions accordingly. In Alabama, where 44% of residents live in rural areas, addressing ACEs through preventive services may present unique challenges [14]. Moreover, Alabama as a southern state has disproportionate rates of preventable diseases of the metabolic triad (obesity, diabetes, and heart disease) compared to more northern states [15].

The Exchange Center for Child Abuse Prevention (ECCAP), a trauma-informed center serving the rural Wiregrass region of Alabama, has been aiming to break the cycle of child abuse and family violence since 1978. Trauma and therapy support programs provided by ECCAP (e.g., Group Trauma Counseling for Sexual Abuse Survivors, Family Counseling, Trauma-Informed Yoga for Teens and Adults, Home Based Parent Aide services for At-Risk Families, Parenting Classes (Houston County and Geneva County), Anger Management Class for Adults, School-Based and Community-Based Prevention Programs, Biofeedback, Trauma-Informed Yoga, and Professional Trainings and In-Services on Trauma-informed Care) are free to their clients and have grown and expanded tremendously in the last 44 years. As a 501 C-3 non-profit organization, the agency is funded through grants, donations, and fundraisers to support its primary prevention programs. ECCAP has served over 50,000 clients referred for services by the local medical community, shelters, social service agencies, state departments, churches, schools, or through self-referral since its inception. Beyond clients, ECCAPs reach in community education events expands to 300,000 individuals. This agency serves both rural counties (Coffee, Dale, Henry, Covington, Geneva, and Pike) and urban counties (Houston) in Alabama's Wiregrass region.

For over five years, ECCAP has been gathering data on ACEs among its clients. This information may guide the development of effective interventions for rural clients with a history of trauma. To gain insights into the characteristics and needs of ECCAP clients, we investigated the following objectives: (i) To compare ACE scores between ECCAP clients from rural and urban counties in Alabama’s Wiregrass region and determine if significant differences exist using the Kruskal-Wallis test. (ii) To analyze the relationship between ACE scores and victimization categories identified at intake. (iii) To assess the general association between ECCAP ACE scores and general metabolic triad outcomes (diabetes, hypertension, and obesity) in Wiregrass counties. (iv) To identify implications for enhancing trauma-informed care and intervention strategies while providing data to support the development of targeted interventions for high-ACE populations in rural areas.

We hypothesized that ECCAP clients in rural Wiregrass counties will have higher ACE scores than those in urban counties and that ACE scores will be directly correlated with the prevalence of metabolic diseases. Additionally, we hypothesized that ACE scores will not differ significantly based on client-presenting victimization.

Despite the presence of other trauma-informed centers in Alabama like the Montgomery Sunshine Center, the research on their effectiveness and the ACE scores of their clients is limited. Indeed, there is no published ACE research on the clientele of any trauma-informed center in the state of Alabama. Hence, collaborating with ECCAP to analyze their data is crucial for improving trauma-informed services in the state. This research can serve as a precedent for data collection and aid in mitigating the long-term effects of toxic stress.

This article was previously presented as a meeting abstract at the Alabama College of Osteopathic Medicine/Society of Hospital Medicine Research Week and Student Osteopathic Association for Research Poster Day held on November 29-December 2, 2022.

## Materials and methods

Survey

The data provided by the Exchange Center spanned three years (2019-2021), utilizing the original Centers for Disease Control and Prevention (CDC)-Kaiser Permanente Adverse Childhood Experiences Questionnaire (ACE-Q) [16]. The ACE-Q was provided to all clients at the Exchange Center during initial intake as an optional survey. In-take presenting victimization codes and related patient information were used based on the Victims of Crimes Act (VOCA) Victim’s Assistance Grant Program [17]. ACE scores served as the independent variable and presenting victimization served as the dependent variable in this study. Both the ACE-Q and VOCA surveys are provided in the Appendices. Once the surveys were completed, each counselor uploaded their respective client’s data into an electronic Excel database (Microsoft Corp., Redmond, US). Though beyond the scope of this work, family history, a wellness assessment, and therapy goals were also gathered during the initial intake and additional post-therapy surveys were periodically given to assess therapeutic outcomes specific to client goals.

Participants

The research team focused on ACE-Q and VOCA data collected from ECCAP clients whose primary residences were from the following Wiregrass counties: Coffee, Covington, Dale, Geneva, Henry, Houston, and Pike. Data collected outside of the defined region were excluded from the study. In addition, any incomplete data were excluded from the study. Of the dataset provided, 237 clients were excluded from analysis due to living outside of areas of interest for a final dataset of N=1643. Due to the anonymous nature of the data, we did not have information on clients' specific past medical history.

Statistics

Statistical analyses were conducted using RStudio IDE open source edition (Posit, Boston, US) [18,19]. Non-parametric tests, the Kruskal-Wallis test, and the Wilcoxon Rank Sum Test were used with the ACE score set as the dependent variable. Dataset and codes are available upon request.

Heat map

Tableau (Salesforce Inc., San Francisco, US) [20] was utilized to develop a heat map depicting average ACE scores across the Wiregrass region. Included ECCAP data were uploaded into Tableau. Utilizing Tableau geolocation features, Wiregrass counties were mapped. Average ACE scores were on a green-to-orange gradient, with green representing the lowest calculated average ACE score (Henry, Coffee) and orange representing the highest calculated ACE score (Covington). The map was downloaded as .pptx with calculated statistics and a zoomed-out Tableau map of the greater southeast region added. Tableau map data were derived from Mapbox (www.mapbox.com) [21].

PolicyMap method

PolicyMap (www.policymap.com) [22] was utilized to find visual population data regarding diabetes, hypertension, and obesity. A layer of US counties was added to the map, and a boundary was drawn around counties where ECCAP collected ACE data. The data for diabetes, hypertension, and obesity was then clipped to display data only within the Wiregrass counties. Map data was derived from CDC Population Level Analysis and Community Estimates (PLACES) data [23]. PLACES uses multilevel regression and poststratification (MRP) modeling to estimate the prevalence of diseases for a given geospatial area. The tabulation area used for the PLACES data was the zip code. The small area estimates displayed in the PolicyMap images were sourced from the CDC’s Behavioral Risk Factor Surveillance System and the National Survey of Children’s Health [24].

## Results

**Figure 1:**
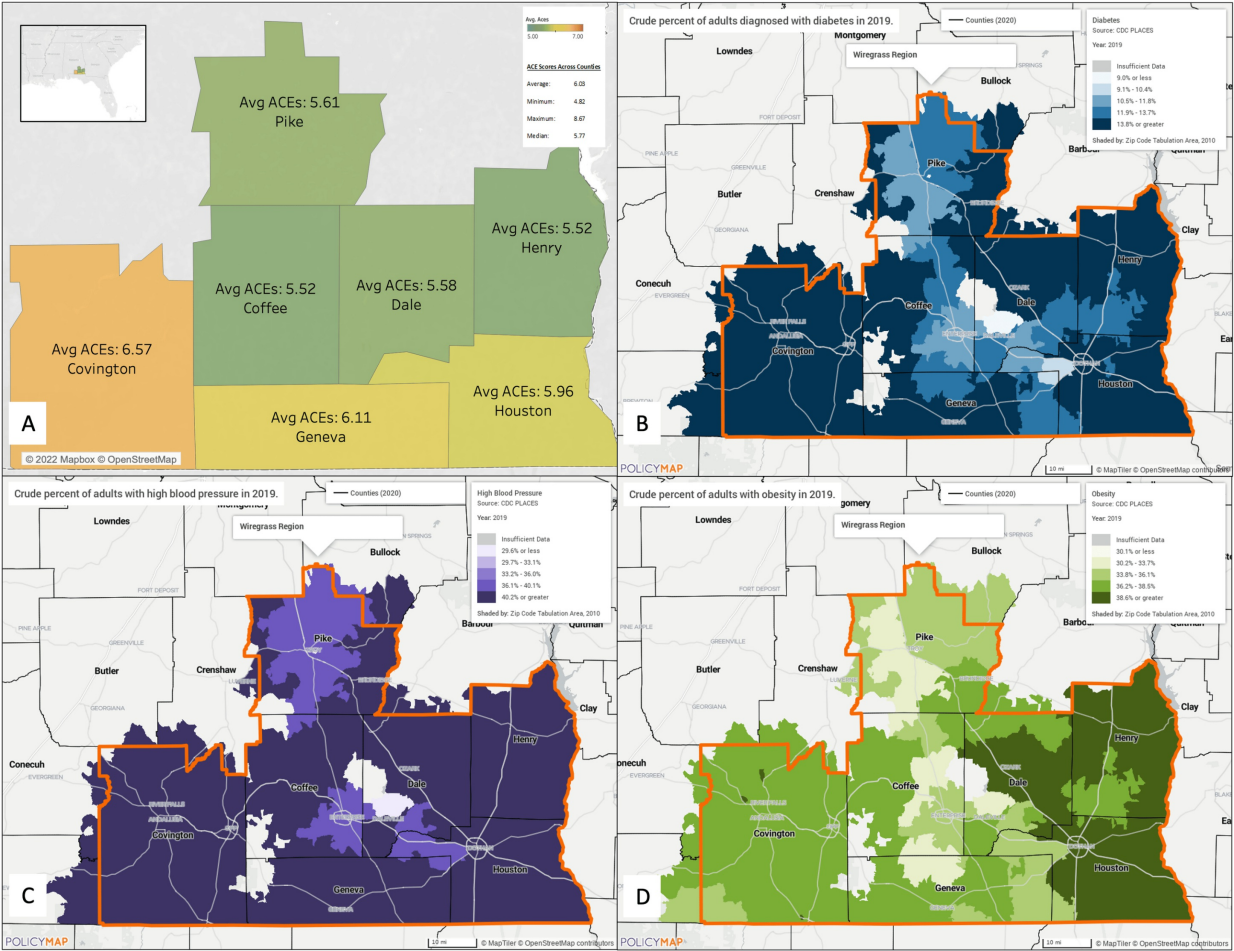
ECCAP client ACE scores and metabolic triad outcomes across Wiregrass counties (A) Average ACE scores of the clients from the Exchange Center in Dothan, Alabama, between 2019 and 2021. The scale ranges from 5 to 7 ACEs. The summary includes the average ACE score for the region, with all seven represented counties having an average score of 6.03. (B) Crude percentage of adults diagnosed with diabetes in 2019. (C) Crude percentage of adults diagnosed with high blood pressure in 2019. (D) Crude percentage of adults diagnosed with obesity in 2019. The maps were created using PolicyMap (www.policymap.com). ECCAP: Exchange Center for Child Abuse Prevention; ACE: Adverse childhood experience

We evaluated how ECCAP ACE scores compare to clients' county of origin based on the rural status of each county in the context of the metabolic triad outcomes of each county. According to the Exchange Center data, an overall mean ACE score (N=1643) of 5.897 across seven Alabama counties in the Wiregrass area was found. There was no significant difference between ACE scores between counties (Kruskal-Wallis Test and Pairwise Wilcoxon Test p>0.05), meaning that clients had similar ACE scores regardless of their county of origin (Kruskal-Wallis Test and Pairwise Wilcoxon Test p>0.05) (Figure 1A). Per the databases and PolicyMap, the metabolic triad health outcomes in Wiregrass counties were surveyed (Figures 1B-1D). The map legends display the ranges for the estimated percentage of people with disease in 2019. As regions of the map darken, the estimated prevalence of disease increases. The diabetes map (Figure 1B) shows that most zip codes have 13.8% or higher percentages of people living with the disease. The hypertension map (Figure 1C) shows that most zip codes have 40.2% or a higher percentage of people living with the disease. The obesity map (Figure 1D) shows that most zip codes have 36.2%-38.5% living with the disease. When compared to each other, hypertension had the highest prevalence in the Wiregrass region, followed by obesity, and diabetes had the lowest prevalence (according to CDC PLACES data, all maps estimated disease prevalence despite inconsistency with layer naming). No direct visual correlation was found between higher ACE scores per county and higher prevalence of metabolic triad health outcomes.

**Figure 2:**
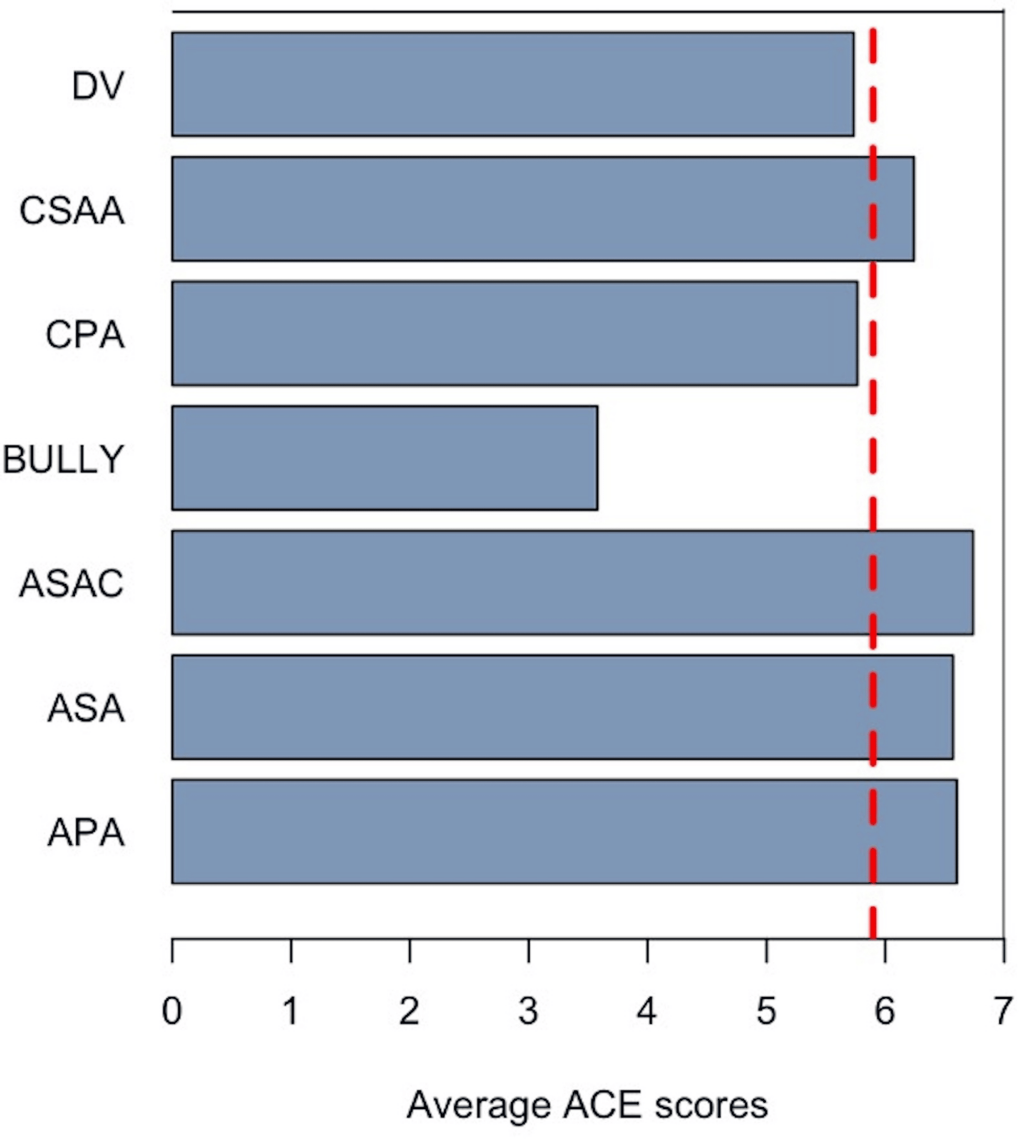
ECCAP ACE scores by victimization The average ACE scores of all EPPAC clients (n=1643) are noted by the red dotted line (5.896531). Categories are as follows: APA - Adult Physical Assault, ASA - Adult Sexual Assault; ASAC - Adult Sexually Abused As Children; BULLY - Verbal, Cyber, or Physical; CPA - Child Physical Abuse or Neglect; CSAA - Child Sexual Abuse or Assault; DV - Domestic Violence or Family Violence. ECCAP: Exchange Center for Child Abuse Prevention; ACE: Adverse childhood experience

Furthermore, we wanted to understand how the ACE scores of clients relate to their VOCA in-take presenting victimization. The first step involved examining the distribution of data. Most clients were those presenting with Domestic Violence (DV) (n=782, 48%) and Child Physical Abuse or Neglect (CPA) (n=464, 28%), followed by Adult Sexually Abused As Children (ASAC) (n=183, 11%) and Child Sexual Abuse or Assault (CSAA) (n=145, 8%). The lowest number (under 5%) of clients presented with Adult Sexual Abuse (ASA) (n=37), Bullying (BULLY) (n=19), and Adult Physical Assault (APA) (n=10). Clients who came to the clinic with the in-patient code ASAC had the highest ACE scores of all clients (6.737705) (Figure 2). Those reporting verbal, cyber, or physical bullying (BULLY) had the lowest ACE scores of all clients (3.578947) (Figure 2).

## Discussion

ACEs represent a national health crisis, with 45% of children in the US experiencing at least one ACE [1]. Decades of research support a strong correlation between childhood exposure to household dysfunction and long-term health risks driven by toxic stress [9-11]. However, there remains a lack of research on the effects of ACEs in medically underserved rural populations, particularly in Alabama’s Wiregrass region. Given that 14% of the US population resides in rural areas, it is essential to examine how ACEs impact these communities and their association with preventable negative health outcomes. Rural populations face unique challenges, including limited access to healthcare, higher rates of poverty, and fewer mental health resources, all of which can compound the effects of childhood adversity. Understanding these regional disparities is critical for tailoring interventions that address both ACE-related trauma and systemic barriers to care.

Client intake data from the Exchange Center (N=1643) between 2019 and 2021 revealed an overall mean ACE score of 5.897 across seven Wiregrass counties, with no significant differences between counties (p>0.05). Thus, the null hypothesis was not rejected, as ACE scores remained consistent across county lines. However, it is important to note that the ECCAP client population is not representative of the broader Wiregrass region. Future studies should incorporate a more diverse sample to capture the full scope of ACE exposure. Additionally, ACE data were categorized by county rather than zip code, limiting the ability to detect localized health disparities. Prior studies have demonstrated that zip code level data when combined with income level can provide more precise insights into health outcomes and intervention needs [25].

To further analyze ACE score variations, we examined differences based on presenting victimization categories from VOCA intake data. As shown in Figure 2, individuals who reported being ASAC had the highest ACE scores, followed closely by those presenting with ASA and APA. This suggests that these groups significantly contribute to the overall high average ACE score. Given that VOCA prioritizes funding for victims of sexual assault, domestic violence, and child abuse, these findings underscore the necessity of continued support for these populations. Conversely, clients presenting with verbal, cyber, or physical bullying had the lowest ACE scores, though their scores still exceeded the national average [1,26]. Notably, ASAC, APA, and ASA clients collectively represented nearly 15% of ECCAP’s client base, highlighting the urgent need for trauma-informed services.

We also explored the relationship between ECCAP client data and broader health trends in the Wiregrass region, hypothesizing that there would be visible correlations between ACE scores and metabolic triad outcomes (diabetes, hypertension, and obesity). Using PolicyMap data derived from CDC PLACES, we surveyed these health outcomes across Wiregrass counties. Among the three conditions, hypertension had the highest prevalence, followed by obesity and diabetes. While extensive research has established links between ACE exposure and metabolic triad diseases, no direct visual correlations between the two were found in our dataset [9-11]. There are two key methodological reasons for this. First, the patient population in the Wiregrass region does not fully align with the population studied in the PLACES data. Second, while PLACES data is analyzed at the zip-code level, ECCAP data is grouped by county, potentially masking granular geographic trends. Future studies should integrate past medical history with ACE survey responses to establish more direct connections between early trauma and long-term health outcomes. In clinical settings, incorporating ACE surveys into in-patient assessments could facilitate real-time referrals to free intervention centers, improving access to trauma-informed care.

While ACE surveys can be useful for guiding clinical interventions, they also have limitations. Some healthcare providers have implemented structured protocols for using ACE surveys during intake to assess patient exposure to toxic stress [27]. However, ACE screening primarily identifies household-related stressors, potentially overlooking external adversities such as community violence or socioeconomic instability, which could present in the rural setting. ECCAP expands upon standard ACE-Q data by incorporating external stressors, providing a more comprehensive assessment of client needs. Research suggests that the most effective ACE intervention programs must account for both past and present stressors, equipping patients and caregivers with strategies to improve their environment and long-term health outcomes [2].

Many aspects of ACE surveys and similar screening tools are beneficial for clinicians to begin creating treatment plans and interventions. Pediatricians are uniquely situated to identify abuse in children and provide resources that may redirect negative stressors to aid children in reaching their physical, social, and emotional potential. Following an ACE survey, effective interventions may include referring the patient to a child psychologist for evidence-based therapies such as cognitive behavioral therapy and providing support and counseling for the patient's caregivers. These interventions can provide positive coping mechanisms for children in a way that is non-traumatizing and promotes growth [28].

Despite their benefits, ACE surveys alone are insufficient for determining long-term health risks, as they fail to assess protective factors such as positive coping mechanisms, access to social support, or community resources. This limitation makes it inadvisable to rely solely on ACE scores when evaluating a patient’s current or future health risks [2]. Moreover, the implementation of ACE surveys must be handled with care, as the screening process itself has the potential to re-traumatize individuals or alter their perception of past abuse. Without clear evidence-based protocols for post-screening interventions, ACE scores alone may not translate into meaningful patient care. Additionally, concerns about mandatory reporting or other perceived threats may discourage patients and caregivers from responding accurately, further complicating data collection. Socioeconomic background and family circumstances also influence how trauma disclosures are managed, including whether referrals to child protective services or specialists occur [29].

Despite these challenges, ACE surveys remain a valuable tool for initiating trauma-informed care. They should be regarded as screening instruments rather than diagnostic tools. To enhance their effectiveness, follow-up assessments should be implemented to track long-term health impacts and identify mitigating factors that support resilience. This information can inform personalized treatment plans that address each patient’s unique needs. ECCAP builds on ACE-Q intake data by providing evidence-based, trauma-informed care tailored to its client population, reinforcing the importance of holistic and responsive intervention strategies, especially for rural Alabamians. 

## Conclusions

The findings from the ECCAP dataset underscore the pervasive impact of ACEs among ECCAP clients in both rural and urban populations of Alabama’s Wiregrass region. The mean ACE score of nearly 6 suggests a high prevalence of childhood trauma among this specific population, highlighting the need for trauma-informed care to mitigate potential long-term health consequences. However, these findings are limited to ECCAP clients, and further research is needed to confirm whether similar patterns exist in the broader Alabamian population. While the data indicate that certain victimization categories, particularly ASAC, correlate with higher ACE scores, the study's cross-sectional design limits causal interpretations. These relationships should be explored further in longitudinal studies to better understand the directional influences between ACEs and health outcomes.

The lack of significant correlations between ACE scores of ECCAP clients and general metabolic triad outcomes (hypertension, obesity, diabetes) in the Wiregrass region of Alabama warrants further investigation. Potential explanations include dataset differences, unmeasured confounding factors, or the need for more granular health data to detect nuanced associations. Future analyses should incorporate more comprehensive health metrics and control for lifestyle and socioeconomic factors that may influence these outcomes. Ultimately, expanding research beyond ECCAP clients and refining trauma-informed policies will be critical in developing targeted, effective interventions that break the cycle of trauma and improve health outcomes in vulnerable communities.

## Appendices

Adverse childhood experience (ACE) survey

1. Did a parent or other adult in the household often… Swear at you, insult you, put you down, or humiliate you? Or Act in a way that made you afraid you might be physically hurt? Yes       No        If yes enter 1             

2. Did a parent or other adult in the household often… Push, grab, slap, or throw something at you? Or Ever hit you so hard that you had marks or were injured? Yes       No        If yes enter 1   

3. Did an adult or person at least 5 years older than you ever… Touch or fondle you or have you touch their body in a sexual way? Or Try to actually have oral, anal, or vaginal sex with you? Yes       No        If yes enter 1              

4. Did you often feel that… No one in your family loved you or thought you were important or special? Or Your family didn’t look out for each other, feel close to each other, or support each other? Yes       No        If yes enter 1                 

5. Did you often feel that… You didn’t have enough to eat, and to wear dirty clothes, and had no one to protect you? Or Your parents were too drunk or high to take care of you or take you to the doctor if you need it? Yes       No        If yes enter 1         

6. Were your parents ever separated or divorced? Yes       No        If yes enter 1         

7. Was your mother or stepmother… Often pushed, grabbed, slapped, or had something thrown at her? Or Sometimes or often kicked, bitten, hit with a fist, or hit with something hard? Or Ever repeatedly hit over at least a few minutes or threatened with a gun or knife? Yes       No        If yes enter 1   

8. Did you live with anyone who was a problem drinker or alcoholic or who used street drugs? Yes       No        If yes enter 1                 

9. Was a household member depressed or mentally ill or did a household member attempt suicide? Yes       No        If yes enter 1                 

10. Did a household member go to prison? Yes       No        If yes enter 1                 

Now add up your “Yes” answers This is your ACE score

Procedures for Victims of Crimes Act (VOCA) database

1. Enter client demographics Last name, First name, Age, Race/Ethnicity, Sex, Date Entered, Date Exited, County

2. Special classification Def - Deaf/Hard of Hearing H - Homeless L - LGBTQ V - Veteran CMPD - Cognitive, Mental, or Physical Disability ESL - Limited English

3. Enter client ACE score

REQUIRED FOR OUTCOME MEASURES

4. Update client phase of healing after 6 months, or post case closure

REQUIRED FOR OUTCOME MEASURE

5. Presenting victimization - what prompted the client to access services, enter code APA - ADULT PHYSICAL ASSAULT ASA - ADULT SEXUAL ASSAULT ASAC - ADULT SEXUALLY ABUSED AS CHILDREN BULLYING - VERBAL, CYBER, OR PHYSICAL CPA/NEG - CHILD PHYSICAL ABUSE OR NEGLECT CP - CHILD PORN CSA/A - CHILD SEXUAL ABUSE OR ASSAULT DV/FV - DOMMESTIC VIOLENCE OR FAMILY VIOLENCE HT/SEX - HUMAN TRAFFICKING/SEXUAL STK/HAR - STALKING/HARASSMENT TDV - TEEN DATING VIOLENCE

6. History of abuse Enter an X in all categories that apply to the client throughout their lifetime APA - ADULT PHYSICAL ASSAULT ASA - ADULT SEXUAL ASSAULT ASAC - ADULT SEXUALLY ABUSED AS CHILDREN BULLYING - VERBAL, CYBER, OR PHYSICAL CPA/NEG - CHILD PHYSICAL ABUSE OR NEGLECT CP - CHILD PORN CSA/A - CHILD SEXUAL ABUSE OR ASSAULT DV/FV - DOMESTIC VIOLENCE OR FAMILY VIOLENCE HT/SEX - HUMAN TRAFFICKING/SEXUAL STK/HAR - STALKING/HARASSMENT TDV - TEEN DATING VIOLENCE

7. Information referral Put an X if client received a referral of any type Enter the number of times the service has been provided for each client. If you referred a client to the DA twice, enter number 2. If you referred a client to the House of Ruth, Alfred Saliba, and Wallace that quarter, enter the number 3, etc.

Types of referrals Information about the criminal justice progress Information about victims’ rights

Referral to other services, supports, and resources (includes legal, faith-based, etc.)

8. Emotional support/safety services Enter the number of times the services has been provided for each client. If you provide Group Therapy 9 times that quarter, enter the number 9. If you provide individual therapy 12 times that quarter, enter the number 12

Types of emotional support/safety services Crisis interventions/safety plan Individual counseling Support group/group therapy Telephone contact Emergency financial assistance
